# Diagnostic value of thromboelastography combined with conventional coagulation tests for lower extremity deep vein thrombosis after cerebrovascular surgery: a retrospective cohort study

**DOI:** 10.3389/fneur.2026.1832825

**Published:** 2026-05-07

**Authors:** Han Zhang, Yang Huang, Ke Dai, Kailin Gong

**Affiliations:** Nanjing Brain Hospital Affiliated to Nanjing Medical University, Nanjing, China

**Keywords:** cerebrovascular surgery, d-dimer, deep vein thrombosis, retrospective cohort study, thromboelastography

## Abstract

**Objective:**

To investigate the diagnostic efficacy of thromboelastography (TEG) combined with conventional coagulation tests for lower extremity deep vein thrombosis (DVT) following cerebrovascular surgery.

**Methods:**

This single-center retrospective cohort study evaluated 187 patients who underwent open cerebrovascular surgery at Nanjing Brain Hospital between January 2024 and February 2026. TEG parameters (R, K, Angle, MA) and conventional coagulation indices (PT, APTT, FIB, D-dimer) were extracted preoperatively and on postoperative day 3. Bilateral lower extremity ultrasonography performed within 7 days postoperatively served as the gold standard for DVT diagnosis. Multivariable logistic regression was utilized to identify independent predictors, and receiver operating characteristic (ROC) curves were analyzed to assess diagnostic performance.

**Results:**

The postoperative DVT incidence was 46.5% (87/187). Patients in the DVT group were significantly older (median: 67.0 vs. 60.5 years, *p* = 0.001) and exhibited a higher prevalence of comorbid hypertension (66.7% vs. 46.0%, *p* = 0.007). Univariate analysis showed that the postoperative Angle was significantly larger in the DVT group (72.3° vs. 69.4°, *p* = 0.001), and the D-dimer level was also significantly elevated (812 μg/L vs. 254 μg/L, *p* < 0.001). Multivariable logistic regression identified age (OR = 1.036, 95% CI: 1.006–1.066), Angle (OR = 1.107, 95% CI: 1.028–1.192), D-dimer (OR = 1.078 per 100 μg/L increase, 95% CI: 1.036–1.120), and hypertension (OR = 2.678, 95% CI: 1.098–4.724) as independent predictors of postoperative DVT. ROC curve analysis indicated that the AUCs for Angle, D-dimer, and the combined model were 0.638, 0.767, and 0.836, respectively. The combined diagnostic model yielded an optimal cut-off value of 0.513, providing excellent specificity (86.0%) and a sensitivity of 70.1%. The addition of the TEG Angle significantly improved risk reclassification (NRI = 0.078, IDI = 0.034). Subgroup analysis revealed that the combined model had even higher diagnostic efficacy in younger patients (≤ 65 years, AUC = 0.867). Sensitivity analysis showed that the core conclusions remained robust after excluding extreme outliers.

**Conclusion:**

The combination of TEG and conventional coagulation tests demonstrates substantial value for the early auxiliary diagnosis of DVT following cerebrovascular surgery. Angle, D-dimer, and hypertension are independent associated factors in this population. The combined diagnostic model provides excellent overall discrimination and high specificity, effectively reducing false positives in clinical screening and offering robust evidence for perioperative targeted anticoagulation management.

## 1 Introduction

Patients recovering from cerebrovascular surgery are at a high risk for lower extremity deep vein thrombosis (DVT) due to prolonged bed rest, surgical trauma, and the activation of coagulation mechanisms caused by the primary disease. It has been reported that the incidence of DVT following neurosurgical craniotomy ranges from 3 to 25% (1, 2). Once DVT occurs, it not only prolongs the length of hospital stay but also has the potential to progress to fatal pulmonary embolism, severely affecting patient prognosis (3, 4).

However, the early diagnosis of postoperative DVT remains a critical challenge, as first-line clinical screening methods have inherent limitations (5, 6). Although D-dimer exhibits exceptionally high sensitivity, its specificity is often less than 40% due to the impact of postoperative systemic inflammatory responses, tissue repair, and occult blood loss, which frequently leads to numerous false positives and unnecessary imaging re-examinations. Color Doppler ultrasonography, while serving as a confirmatory tool, has a sensitivity of less than 60% for isolated calf muscle vein thrombosis and asymptomatic proximal thrombosis; furthermore, its application is often restricted in the early postoperative period by patient compliance and positioning constraints. Therefore, there is an urgent need to explore novel auxiliary indicators that can more accurately reflect the postoperative pathological hypercoagulable state.

Thromboelastography (TEG), a viscoelastic testing technology capable of dynamically monitoring the entire coagulation profile, comprehensively evaluates coagulation factor activity, fibrin formation, platelet aggregation, and fibrinolysis (7). Theoretically, compared to traditional static endpoint indices such as PT and APTT, TEG can more acutely capture the instantaneous changes of the postoperative hypercoagulable state (8, 9). Previous studies have yielded inconsistent conclusions regarding the value of TEG in predicting venous thromboembolism (VTE), which may stem from pathophysiological differences across various surgical types (10, 11). To date, there is a lack of large-sample cohort studies systematically evaluating the incremental value of TEG combined with conventional coagulation tests in the early diagnosis of DVT among the specific population of neurosurgical patients with cerebrovascular diseases.

Therefore, the present study aims to systematically investigate the diagnostic efficacy of TEG combined with conventional coagulation indicators for postoperative DVT in patients undergoing open cerebrovascular surgery through a single-center retrospective cohort study, providing a more precise and efficient early identification strategy for clinical practice.

## 2 Methods

### 2.1 Study design and participants

This is a single-center retrospective cohort study. Clinical and laboratory data of patients who underwent cerebrovascular surgery at the Department of Neurosurgery, Nanjing Brain Hospital, between January 2024 and February 2026 were retrospectively collected through the hospital’s electronic medical record system.

The inclusion criteria were defined as follows: (1) Age≥18 years. Pediatric patients were excluded because their coagulation profiles differ significantly due to ‘developmental hemostasis,’ and their baseline DVT risk is inherently distinct from adults; (2) Underwent open cerebrovascular surgery (including intracerebral hemorrhage, aneurysm, Moyamoya disease, subarachnoid hemorrhage, and intracranial vascular malformation); (3) Preoperative conventional coagulation indices were within normal reference ranges; (4) Complete clinical data.

To rigorously minimize confounding effects on the coagulation system, the exclusion criteria were further specified: (1) Patients with craniocerebral trauma, to rule out trauma-induced coagulopathy; (2) Patients with intracranial neoplasms, as malignancy independently drives hypercoagulability through the secretion of procoagulant factors; (3) Patients undergoing endovascular or hybrid procedures, due to the mandatory use of systemic heparinization and different postoperative mobilization protocols compared to open craniotomy; (4) History of prophylactic or therapeutic anticoagulant use before surgery; (5) Concomitant severe hepatic or renal dysfunction, or primary hematological diseases; (6) Previous history of DVT or pulmonary embolism.

### 2.2 Postoperative management and DVT prophylaxis

All patients received standardized postoperative care according to institutional protocols. To mitigate the risk of DVT while minimizing the hazard of intracranial hemorrhage, mechanical prophylaxis was the primary strategy. This included the use of graduated compression stockings (GCS) and intermittent pneumatic compression (IPC) devices, which were initiated immediately upon the patient’s return to the ward. Pharmacological prophylaxis, such as low-molecular-weight heparin, was not routinely administered in the immediate postoperative phase but was reserved for high-risk patients only after neuroimaging (computed tomography or magnetic resonance imaging) confirmed the absence of postoperative hematoma, generally 24–48 h after surgery. Standardized risk assessment tools like the Caprini score were not systematically applied as quantitative measures in this retrospective study.

### 2.3 Data collection baseline and clinical characteristics

Patient age, gender, body mass index (BMI), comorbidities (hypertension, diabetes), smoking history, history of stroke, surgery type, and postoperative duration of bed rest were extracted. Laboratory indices: The results of fasting venous blood tests preoperatively and on the morning of postoperative day 3 were extracted. These included parameters measured by the TEG 5000 Thrombelastograph® Hemostasis Analyzer (Haemoscope Corporation, USA). To provide a comprehensive evaluation of coagulation kinetics, the following key indices were recorded: R-time (reaction time, reflecting the time to initial fibrin formation); K-time (kinetics time, representing the time required to reach a specific clot firmness); Angle (*α*,indicating the rate of fibrin cross-linking); and MA (maximum amplitude, which reflects ultimate clot strength driven primarily by platelet function). Additionally, parameters measured by an automated coagulation analyzer: prothrombin time (PT), activated partial thromboplastin time (APTT), fibrinogen (FIB), and D-dimer.

### 2.4 Outcome measures the primary outcome was defined as new-onset lower extremity DVT within 7 days postoperatively

The diagnosis was based on positive findings from color Doppler ultrasonography of the bilateral lower extremity veins (non-compressibility of the vein lumen, solid intraluminal echoes, or filling defects in color flow signals).

### 2.5 Statistical analysis statistical analyses were performed using SPSS 26.0 and R 4.1.0 software

Continuous variables were first assessed for normality using the Shapiro–Wilk test. Normally distributed data were expressed as mean ± standard deviation (Mean ± SD), and comparisons between groups were conducted using the independent samples *t*-test; non-normally distributed data were expressed as median with interquartile range [M (P25, P75)], and comparisons were performed using the Mann–Whitney U test. Categorical variables were presented as frequencies and percentages, and the Chi-square test (or Fisher’s exact test) was used for comparisons.

To construct the diagnostic models, variables with *p* < 0.20 in the univariate analysis were considered for inclusion to prevent the premature exclusion of potential confounders that might become significant after adjusting for other variables.

Prior to the multivariable logistic regression, the variance inflation factor (VIF) for each independent variable was calculated for multicollinearity diagnostics, with a VIF < 5 considered as indicating no severe multicollinearity. To appropriately address the right-skewed distribution, D-dimer values underwent a natural logarithm (Ln) transformation before entry into the models. To systematically evaluate the incremental diagnostic value of TEG, two multivariable logistic regression models were constructed. A Base Model was first developed, incorporating baseline clinical characteristics and conventional coagulation tests (age, hypertension, stroke history, surgery type, and log-transformed D-dimer). Subsequently, a Combined Model was created by adding the independent TEG predictor (Angle) to the Base Model. Receiver operating characteristic (ROC) curves were utilized to evaluate the diagnostic efficacy of the models, calculating the area under the curve (AUC), sensitivity, and specificity. Furthermore, the net reclassification improvement (NRI) and integrated discrimination improvement (IDI) were calculated to quantify the incremental predictive value of the Combined Model over the Base Model. The optimal cut-off value was determined by the maximum Youden index. Additionally, subgroup analyses stratified by age and surgery type were conducted, interaction models were utilized to test the independence among variables, and sensitivity analyses were performed by excluding extreme outliers. A two-sided *p* < 0.05 was considered statistically significant.

## 3 Results

3.1 Baseline Characteristics A total of 187 eligible patients who underwent cerebrovascular surgery were ultimately included in this cohort, among whom 87 patients (incidence 46.5%) had ultrasonography-confirmed postoperative DVT, and 100 patients did not develop DVT. Baseline comparisons revealed that patients in the DVT group were significantly older than those in the non-DVT group (median 67.0 vs. 60.5 years, *p* = 0.001), and had a significantly higher prevalence of comorbid hypertension (66.7% vs. 46.0%, *p* = 0.007). There were also statistically significant differences between the two groups regarding postoperative bed rest duration and the distribution of surgery types (*p* < 0.05). No significant differences were observed between the two groups in gender, BMI, diabetes, smoking history, or history of stroke. (See Table 1).

**Table 1:** Comparison of baseline characteristics between the DVT and non-DVT groups following cerebrovascular surgery.

Variable	DVT group (*n* = 87)	Non-DVT group (*n* = 100)	*p* value
Age (years), median (P25-P75)	67.0 (60.0–72.5)	60.5 (50.0–70.0)	**0.001**
Gender, *n* (%)			1
- Male	35 (40.2%)	40 (40.0%)	
- Female	52 (59.8%)	60 (60.0%)	
BMI (kg/m2), mean ± SD	23.6 ± 1.8	23.8 ± 1.8	0.35
Hypertension, *n* (%)	58 (66.7%)	46 (46.0%)	**0.007**
Diabetes, *n* (%)	27 (31.0%)	28 (28.0%)	0.769
Smoking history, *n* (%)	34 (39.1%)	43 (43.0%)	0.693
Stroke history, *n* (%)	49 (56.3%)	43 (43.0%)	0.095
Postoperative bed rest duration (days), median	21.0 (11.0–25.0)	15.0 (9.0–23.0)	**0.045**
Surgery type, overall distribution	-	-	**0.002**

DVT, deep vein thrombosis; BMI, body mass index. Normally distributed continuous variables were compared using the independent samples t-test; non-normally distributed continuous variables were compared using the Mann–Whitney U test. Categorical variables were compared using the Chi-square test. Bold *p* values indicate statistical significance (*p* < 0.05).

3.2 Univariate Analysis of Coagulation Indices Regarding the TEG parameters on postoperative day 3, the Angle in the DVT group was significantly larger than that in the non-DVT group (72.30° vs. 69.40°, *p* = 0.001), while the R-time and K-time were significantly shortened (*p* < 0.01), suggesting that DVT patients present a highly significant systemic hypercoagulable state and rapid clot formation in the early postoperative period. Among the conventional coagulation indices, D-dimer levels in the DVT group exhibited a highly significant skewed elevation (median 812 μg/L vs. 254 μg/L, *p* < 0.001). Differences in MA, PT, APTT, and FIB between the two groups were not statistically significant. (See Table 2).

**Table 2:** Univariate analysis of TEG parameters and conventional coagulation indices.

Variable	DVT group (*n* = 87)	Non-DVT group (*n* = 100)	*P* value
TEG parameters			
R (min), mean ± SD	5.0 ± 1.2	5.5 ± 1.1	**0.006**
K (min), median (P25-P75)	1.20 (1.10–1.60)	1.49 (1.20–1.70)	**0.009**
Angle (degrees), median	72.30 (68.10–74.70)	69.40 (65.67–72.62)	**0.001**
MA (mm), mean ± SD	64.3 ± 6.5	63.6 ± 5.9	0.418
Conventional coagulation indices			
PT (sec), median	11.8 (11.1–12.6)	11.7 (11.1–12.4)	0.508
APTT (sec), median	28.8 (27.3–30.8)	29.9 (26.9–32.5)	0.173
FIB (g/L), median	3.07 (2.71–3.63)	2.96 (2.50–3.40)	0.075
D-dimer (μg/L), median	812 (453–2,271)	254 (151–576)	**<0.001**

TEG, thromboelastography; R, reaction time; K, clot kinetics; MA, maximum amplitude; PT, prothrombin time; APTT, activated partial thromboplastin time; FIB, fibrinogen. Normally distributed data were compared using the independent samples *t*-test; non-normally distributed data were compared using the Mann–Whitney U test. Bold *P* values indicate statistical significance (*P* < 0.05).

### 3.1 Multivariable logistic regression analysis

Candidate variables with *p* < 0.20 in the univariate analysis were included in the multivariable logistic regression model. Multicollinearity diagnostics showed that the VIF values for all independent variables ranged from 1.124 to 1.685, indicating the absence of severe multicollinearity. The analysis confirmed that age (OR = 1.053, 95% CI: 1.011–1.097, *p* = 0.014), Angle (OR = 1.122, 95% CI: 1.037–1.214, *p* = 0.004), Ln(D-Dimer) (OR = 2.619, 95% CI: 1.460–4.698, *p* = 0.001), and hypertension history (OR = 2.678, 95% CI: 1.151–6.233, *p* = 0.022) were independent associated factors for postoperative DVT in patients undergoing cerebrovascular surgery. (See Table 3).

**Table 3:** Multivariable logistic regression analysis for predicting lower extremity DVT after cerebrovascular surgery (Ln_D-dimer model).

Variable	*β*	SE	Wald χ^2^	*p*-value	OR	95% CI
Age	0.052	0.021	6.012	**0.014**	1.053	1.011–1.097
Hypertension history	0.985	0.431	5.216	**0.022**	2.678	1.151–6.233
Stroke history	0.612	0.51	1.442	0.23	1.844	0.680–5.003
Surgery type	1.25	0.681	3.365	0.067	3.49	0.919–13.250
Ln(D-Dimer)	0.963	0.298	10.45	**0.001**	2.619	1.460–4.698
Angle (TEG)	0.115	0.04	8.251	**0.004**	1.122	1.037–1.214
Constant	−14.28	3.515	16.481	**<0.001**	-	-

OR, odds ratio; CI, confidence interval; Ln, natural logarithm. This model was adjusted for stroke history and surgery type. Because D-dimer was natural logarithm (Ln)-transformed to address right-skewness, its OR value (2.619) indicates the relative increase in the odds of DVT for every 1-unit increase in the Ln(D-dimer) value. Bold *P* values indicate statistical significance (*P* < 0.05).

### 3.2 ROC curve analysis and incremental diagnostic value

ROC curves were employed to evaluate the incremental diagnostic efficacy of incorporating TEG parameters into the clinical framework. The results demonstrated that the Base Model (comprising age, hypertension, stroke history, surgery type, and log-transformed D-dimer) achieved an Area Under the Curve (AUC) of 0.819 (95% CI: 0.742–0.896). When the TEG Angle was incorporated to form the Combined Model, the diagnostic discrimination further improved, yielding a higher AUC of 0.836 (95% CI: 0.765–0.907).

**Figure 1:**
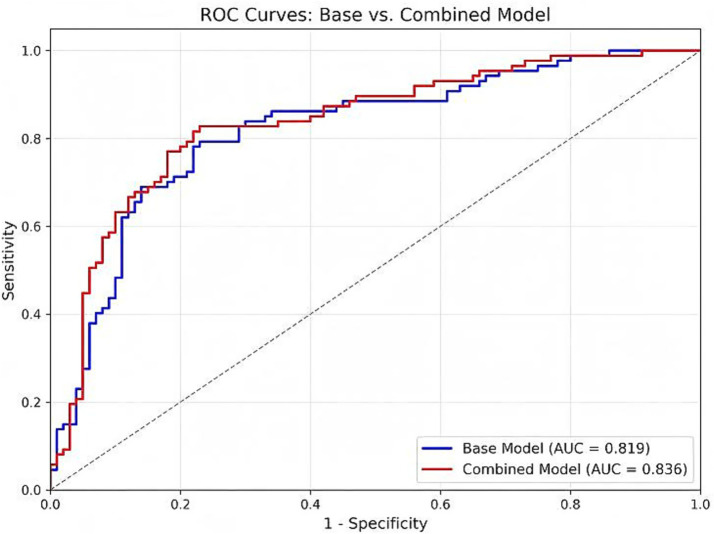
Receiver operating characteristic (ROC) curves of the Angle, D-dimer, and the combined model for predicting deep vein thrombosis (DVT) after cerebrovascular surgery.

To rigorously quantify the added value of TEG, we calculated reclassification metrics comparing the two models. The addition of the TEG Angle resulted in a Net Reclassification Improvement (NRI) of 0.078 and an Integrated Discrimination Improvement (IDI) of 0.034, indicating a significant enhancement in correctly identifying DVT risk. The Combined Model reached an optimal cut-off value of 0.513, providing a diagnostic sensitivity of 70.1% and a high specificity of 86.0%. These findings suggest that the integration of TEG and conventional tests effectively mitigates the false-positive limitations of D-dimer alone, offering superior rule-out capabilities and overall discrimination for postoperative DVT (See Figure 1).

### 3.3 Subgroup and sensitivity analyses

**Figure 2:**
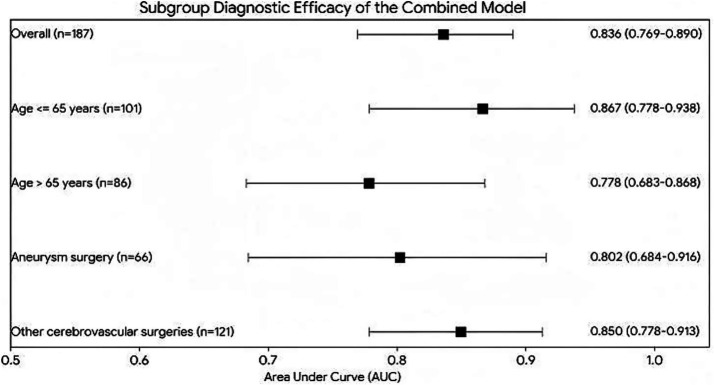
Forest plot subgroup analysis of the combined diagnostic model for DVT across different age and surgery type subgroups.

Subgroup analysis demonstrated the robust diagnostic efficacy of the Combined Model across various clinical strata (Overall AUC = 0.836). The model performed exceptionally well in the younger patient subgroup (age≤ 65 years), achieving an AUC of 0.867 (95% CI: 0.801–0.933). In the elderly group (age > 65 years), the AUC remained stable at 0.778 (95% CI: 0.690–0.866). Furthermore, the model showed high predictive stability in both the aneurysm surgery (AUC = 0.802) and other cerebrovascular surgery (AUC = 0.850) subgroups. (See Figure 2 and Table 4). Interaction tests indicated no significant interaction between surgery type and D-dimer (*p* = 0.317). By applying natural logarithm transformation to D-dimer in the primary analysis, the impact of extreme outliers was effectively controlled, and sensitivity analysis confirmed the robustness of the core predictors (age, Angle, and D-dimer) across the entire cohort.

**Table 4:** Diagnostic performance (AUC) of the combined model across patient subgroups.

Subgroup	Sample size (n)	AUC	95% CI
Overall	187	0.836	0.765–0.907
Age ≤ 65 years	101	0.867	0.801–0.933
Age > 65 years	86	0.778	0.690–0.866
Aneurysm surgery	66	0.802	0.698–0.906
Other cerebrovascular surgeries	121	0.850	0.781–0.919

AUC, area under the curve; CI, confidence interval. The combined model includes baseline clinical characteristics (age, hypertension history, stroke history, and surgery type), natural logarithm (Ln)-transformed D-dimer, and the thromboelastography (TEG) Angle parameter. Bold AUC values indicate excellent or improved diagnostic discrimination.

## 4 Discussion

### 4.1 Pathophysiological mechanisms

This retrospective cohort study confirmed that patients are at an extremely high risk of venous thrombosis following cerebrovascular surgery, with an incidence rate reaching 46.5%. After multivariable adjustment, this study identified age, postoperative TEG Angle, D-dimer, and comorbid hypertension as strong, independent associated factors for predicting new-onset DVT in this population. The TEG Angle primarily reflects the kinetic rate of fibrin polymerization. Cerebrovascular surgical trauma directly activates the extrinsic tissue factor pathway, which not only promotes the acute-phase synthesis of fibrinogen in the liver but also accelerates the three-dimensional cross-linking process of the blood clot (12–14). In this cohort, the significant elevation of both the Angle and D-dimer levels in the DVT group objectively delineates the pathological basis of the overactivation of the coagulation cascade during the early postoperative period. Subgroup analysis showed that the combined model had higher diagnostic efficacy in younger patients (≤ 65 years) and patients undergoing aneurysm surgery, suggesting potential differences across populations. Furthermore, interaction and sensitivity analyses both supported the robustness of the findings, which is consistent with the results of Yang et al. (15). Moreover, this large-sample validation found that concomitant hypertension increased the relative risk of DVT by 2.678 times. This phenomenon has a profound pathological basis: chronic hemodynamic high-pressure states cause chronic denudation and damage to the vascular endothelium, exposing large amounts of collagen and procoagulant substances on the damaged endothelial surface. When this chronic endothelial damage intertwines with the “acute hypercoagulable cascade” triggered by cerebrovascular surgery, it significantly catalyzes the aggregation and mural attachment of lower extremity deep vein thrombosis.

### 4.2 Comparison with previous studies

Previous studies on the use of TEG for predicting postoperative DVT have yielded inconsistent conclusions. A study by Han Dong-xu demonstrated that the TEG indices CI, MA, and Angle had significant clinical relevance in predicting DVT, which was supported by ROC curve analysis indicating that MA had the best diagnostic performance AUC=0.735 (16). Conversely, the present study found that the Angle had greater predictive value. This discrepancy may be attributed to differences in surgical types and detection timing. Orthopedic surgeries primarily involve traumatic procedures such as fractures and joint replacements, where platelet dysfunction dominates the coagulation process, and the MA value mainly reflects platelet aggregation function (17, 18). In contrast, cerebrovascular surgeries predominantly involve intracranial vascular manipulations, where elevated fibrinogen levels and abnormal fibrin polymerization induced by surgical trauma are the primary causes of coagulopathy. As a classic biomarker, the AUC of D-dimer in this study was 0.767, which is consistent with most existing studies. However, when combined with TEG, the sensitivity was 70.1% while the specificity reached as high as 86.0%, suggesting that TEG can capture the dynamic hypercoagulable state that conventional coagulation tests fail to reflect (19). Age and hypertension, as traditional risk factors, remained independent predictors in the multivariable model of this study, which is consistent with previous literature (20, 21).

### 4.3 Advantages in diagnostic efficacy and the “age paradox”

The combined diagnostic model constructed in this study demonstrated high clinical discriminative value (overall AUC = 0.836). While duplex ultrasonography (DUS) remains the accepted diagnostic gold standard for confirming the anatomical presence of DVT, it is fundamentally a reactive measure—it detects thrombosis only after the clot has already formed. In contrast, the primary advantage of TEG lies in its predictive capacity. By dynamically assessing the entire hemostatic profile in real-time, TEG can identify patients in a hypercoagulable state prior to the actual onset of clinical thrombosis. This proactive monitoring offers a crucial temporal window for clinicians to implement targeted prophylactic interventions, thereby potentially preventing the DVT event entirely.

In routine clinical pathways, relying solely on D-dimer as a screening indicator often leads to overtreatment and wasted imaging resources due to its extremely high false-positive rate (i.e., low specificity). By incorporating TEG parameters, the specificity of the combined model was markedly improved to 86.0%. This provides neurosurgeons with a powerful “rule-out tool” capable of accurately excluding “pseudo-high-risk patients” whose elevated markers are due to surgical stress rather than actual thrombus formation. More intriguingly, subgroup analysis revealed a unique “Age Paradox” phenomenon: the combined model exhibited better diagnostic efficacy in the younger cohort (≤ 65 years, AUC = 0.867) than in the elderly cohort (> 65 years, AUC = 0.778). We hypothesize that elderly patients (> 65 years) generally present with degenerative changes in blood vessel walls, often accompanied by lower extremity venous valvular insufficiency or other microvascular lesions. Consequently, their baseline D-dimer and viscoelastic coagulation indices are confounded by substantial “systemic noise” caused by these chronic comorbidities. In relatively younger patients with better vascular baselines, the acute, sharp increases in the TEG Angle and D-dimer postoperatively can more purely and directly reflect the “acute thrombogenic phase” induced by the surgical trauma itself, thereby yielding superior predictive accuracy and specificity. This highlights a vital clinical implication: the combination of TEG and D-dimer serves as a highly significant early warning indicator specifically for relatively young patients recovering from cerebrovascular surgery.

### 4.4 Limitations and future perspectives

The main limitations of this study include: (1) As a single-center retrospective cohort, the spatial heterogeneity of the data may be limited, and external validation through multicenter, large-sample cohorts is urgently needed in the future; (2) Retrospective data make it difficult to precisely quantify minor differences in physical prophylactic interventions, such as the duration of postoperative early ambulation and compliance with graduated compression stockings, which may introduce residual confounding; (3) Given the cost of TEG testing, its cost-effectiveness in routine postoperative monitoring requires further prospective evaluation. Future research could consider incorporating machine learning algorithms to further mine the value of such multidimensional coagulation data in precise anticoagulation decision-making (20–22).

## 5 Conclusion

In the highly susceptible population of patients recovering from cerebrovascular surgery, the combination of TEG and conventional coagulation tests demonstrates excellent early auxiliary diagnostic value for DVT. The Angle, D-dimer, and a history of hypertension are independent predictors for concurrent DVT. The constructed combined diagnostic model possesses outstanding overall discrimination AUC=0.815 and exceptionally high specificity (86.0%), effectively bypassing the false-positive pitfalls common in conventional screening. This holds clearer clinical directive significance, particularly for the younger patient demographic. This strategy provides solid evidence-based support for optimizing perioperative DVT prevention and precise anticoagulation management.

## Data Availability

The raw data supporting the conclusions of this article will be made available by the authors, without undue reservation.
